# KSHV Reactivation from Latency Requires Pim-1 and Pim-3 Kinases to Inactivate the Latency-Associated Nuclear Antigen LANA

**DOI:** 10.1371/journal.ppat.1000324

**Published:** 2009-03-06

**Authors:** Fang Cheng, Magdalena Weidner-Glunde, Markku Varjosalo, Eeva-Marja Rainio, Anne Lehtonen, Thomas F. Schulz, Päivi J. Koskinen, Jussi Taipale, Päivi M. Ojala

**Affiliations:** 1 Genome-Scale Biology Program, Institute of Biomedicine, Biomedicum Helsinki, University of Helsinki, Helsinki, Finland; 2 Institute of Virology, Hannover Medical School, Hannover, Germany; 3 Department of Molecular Medicine, National Public Health Institute (KTL), Helsinki, Finland; 4 Turku Centre for Biotechnology, BioCity, Turku, Finland; 5 Department of Biology, University of Turku, Turku, Finland; 6 The Foundation for the Finnish Cancer Institute, Finland; Oregon Health & Science University, United States of America

## Abstract

Host signal-transduction pathways are intimately involved in the switch between latency and productive infection of herpes viruses. As with other herpes viruses, infection by Kaposi's sarcoma herpesvirus (KSHV) displays these two phases. During latency only few viral genes are expressed, while in the productive infection the virus is reactivated with initiation of extensive viral DNA replication and gene expression, resulting in production of new viral particles. Viral reactivation is crucial for KSHV pathogenesis and contributes to the progression of KS. We have recently identified Pim-1 as a kinase reactivating KSHV upon over-expression. Here we show that another Pim family kinase, Pim-3, also induces viral reactivation. We demonstrate that expression of both Pim-1 and Pim-3 is induced in response to physiological and chemical reactivation in naturally KSHV-infected cells, and we show that they are required for KSHV reactivation under these conditions. Furthermore, our data indicate that Pim-1 and Pim-3 contribute to viral reactivation by phosphorylating the KSHV latency-associated nuclear antigen (LANA) on serine residues 205 and 206. This counteracts the LANA–mediated repression of the KSHV lytic gene transcription. The identification of Pim family kinases as novel cellular regulators of the gammaherpesvirus life cycle facilitates a deeper understanding of virus–host interactions during reactivation and may represent potential novel targets for therapeutic intervention.

## Author Summary

The switch from latency to productive viral replication (reactivation) is a crucial decision in the viral life cycle, and recent clinico-epidemiological studies support the importance of lytic replication in the development and progression of Kaposi's sarcoma. Hence, cellular signaling pathways operative during viral reactivation could represent potential novel targets for therapeutic intervention. Our work identifies Pim-1 and Pim-3 kinases as essential key regulators of the gammaherpesvirus life cycle. These kinases target the hallmark of KSHV latency, the LANA protein, by phosphorylation, which abolishes its ability to act as a transcriptional suppressor of viral lytic replication. This study facilitates a deeper understanding of virus–host interactions during reactivation and provides novel opportunities for pharmacological control and intervention also in virus-associated cancers.

## Introduction

Kaposi's sarcoma herpesvirus (KSHV) is an etiological agent of three types of malignancies: Kaposi's sarcoma (KS), multicentric Castleman disease, and primary effusion lymphoma (PEL) [1]. The KSHV genome encodes homologs of cellular proteins, which deregulate signaling pathways, govern cell proliferation, and apoptosis [2]. KSHV infection displays two different phases: latent and lytic. Most tumor cells are latently infected [3],[4], and the viral genome remains episomal with only few viral genes expressed [5]. Upon induction of the lytic phase, extensive viral DNA replication is initiated leading to expression of viral lytic genes (reactivation), and production of new infectious viral particles [6]. Viral reactivation is important for spreading of progeny virions between cells and hosts, and is critical for the progression to KS [7],[8].

Latency-associated nuclear antigen (LANA) encoded by the open reading frame 73 (ORF73) of the KSHV genome is expressed in all KSHV-infected cells [9],[10]. During latency, LANA tethers the viral episomal DNA to the host chromosomes upon cell division [11],[12]. Many gammaherpesviruses such as KSHV, Epstein-Barr virus (EBV), rhesus rhadinovirus, herpesvirus saimiri, and murine herpesvirus 68 encode a replication and transcription activator protein (RTA) which plays a critical role in the initiation of viral lytic gene expression (reviewed in [13]). RTA is a transcription factor that activates expression of multiple downstream target genes through the RTA-responsive elements and also autoregulates its own promoter. Expression of RTA is necessary and sufficient to disrupt latency and trigger the complete lytic cascade. LANA also represses expression of RTA and other RTA-responsive lytic genes [14].

Multiple cellular signaling pathways have been shown to regulate KSHV reactivation. Hypoxia and inflammatory cytokines including interferon-γ and oncostatin M [15],[16],[17],[18] induce KSHV reactivation, but the underlying mechanisms remain undefined. The primary target of the Notch signaling pathway, RBP-Jκ, mediates RTA-dependent activation of KSHV lytic genes [19], and constitutive activation of Notch1 via over-expression of its intracellular domain is sufficient to reactivate KSHV from latency in PEL cells [20]. Moreover, recent reports further imply that Ras/Raf/MEK/ERK/Ets-1, JNK and p38 MAPK pathways mediate TPA-induced KSHV reactivation [21],[22],[23]. Many chemicals can also induce reactivation in cell culture. These include 12-O-tetradecanoyl-phorbol-13-acetate (TPA) and histone deacetylase inhibitors such as sodium butyrate (NaB) or trichostatin A (reviewed in [24]).

To allow unbiased genome-wide analysis of cross-talk between cellular kinase pathways and KSHV reactivation, we recently carried out a gain-of-function screen utilizing a novel expression library for human protein kinase cDNAs [25]. The screen assessed the ability of 480 individual, ectopically expressed human kinases to induce KSHV reactivation, and identified Pim-1 as a novel kinase involved in KSHV reactivation.

Pim-1 belongs to an oncogenic serine/threonine kinase family with two other members, Pim-2 and Pim-3, sharing significant sequence similarities and largely overlapping functions with Pim-1 (reviewed in [26],[27]). Pim kinases are overexpressed in various lymphomas and leukemias [28],[29] as well as in prostate cancer [30]. Recent results have implicated Pim kinases in regulation of herpesviral oncogenesis. Expression levels of Pim-1 and Pim-2 are up-regulated upon EBV infection and they in turn enhance the activity of the viral nuclear antigen EBNA2, suggesting roles in EBV-induced immortalization and tumorigenesis [31]. In addition, KSHV infection has been shown to enhance expression of Pim-2 in CD34+ bone marrow cells [32].

In this report, we have analyzed the requirement of Pim family kinases in the induction of viral reactivation and investigated the underlying molecular mechanism. Our results demonstrate that Pim-1 and -3 are required for KSHV reactivation, and that phosphorylation of LANA by Pim-1 and -3 counteracts LANA-dependent repression of viral transcription. These results implicate Pim-1 and -3 as critical regulators of KSHV reactivation.

## Results

### Ectopic expression of Pim kinases induces KSHV reactivation

**Figure 1:**
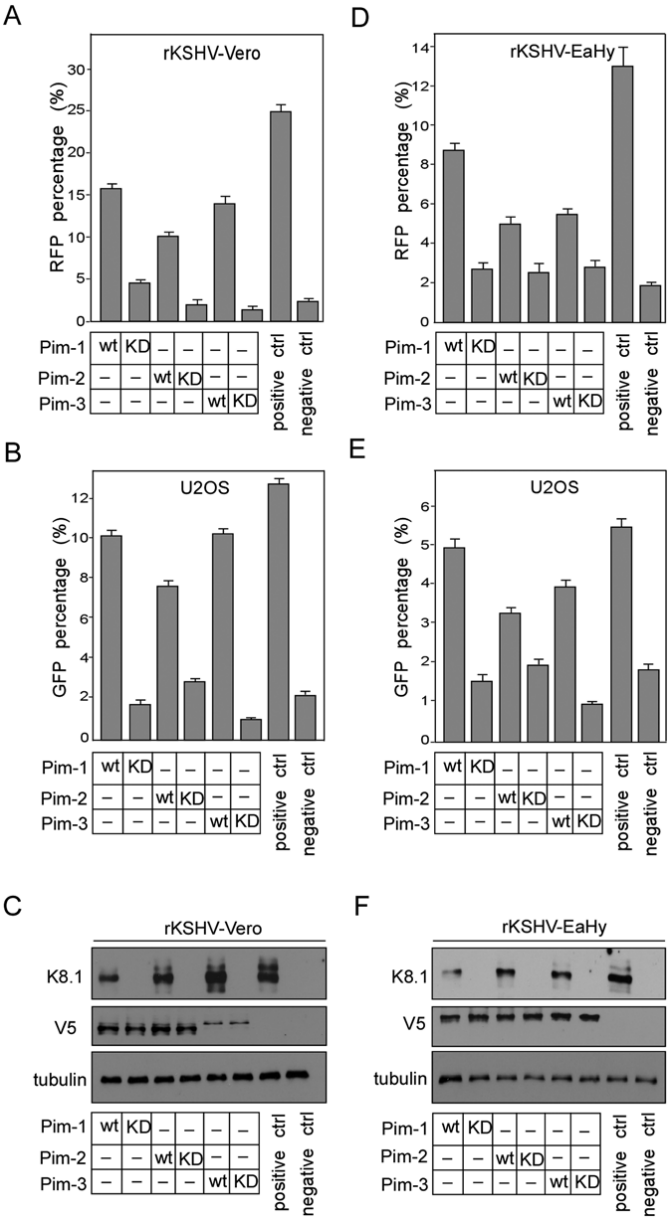
All three Pim kinases are able to induce KSHV reactivation. (A) rKSHV.219–infected Vero cells (rKSHV-Vero) or (D) EA.hy926 cells (rKSHV-Ea.hy) were transfected with the V5-tagged expression vectors for wt and kinase-deficient (KD) forms of Pim kinases as indicated and infected with RTA baculovirus 48 h after transfection (negative control). NaB and infection with RTA baculovirus were used to induce maximal reactivation (positive control). Empty vector transfection was used in the negative and positive controls. 30 h later, the cells were analyzed for RFP expression. 96 h after adding RTA or RTA and NaB, the supernatants from KSHV-Vero (B) or KSHV-EA.hy926 (E) cells were collected and used to infect U2OS target cells. 72 h after infection, U2OS cells were analyzed for GFP expression (a measure of production of infectious virions). Values are means of two independent experiments ±SD. 48 h after addition of RTA, cell extracts from rKSHV-Vero (C) or rKSHV-EA.hy926 (F) were analyzed by Western blotting with antibodies against lytic protein K8.1 and the V5-tag on Pim kinases. Anti-tubulin served as a loading control.

Our recent gain-of function kinome screen identified Pim-1 as a novel kinase inducing KSHV reactivation upon over-expression [25]. In the screen, a genome-wide collection of protein-kinase cDNAs was transfected to Vero cells latently infected with a recombinant KSHV (rKSHV.219 [33]). The rKSHV.219 is a double-reporter virus, which expresses the green fluorescent protein (GFP) from the constitutively active EF-1α-promoter, and can be induced to express the red fluorescent protein (RFP) from the KSHV transactivator protein (RTA)-responsive lytic promoter for polyadenylated nuclear RNA (PAN). PAN is the most abundant transcript made during the lytic phase [34]. Viral reactivation was screened by analysis of RFP-positive cells using an automated high-content microscope. As Pim-1 is not the only representative of its kinase family, we decided to examine the roles of other Pim kinase family members in viral reactivation using the rKSHV.219-Vero cells. The experiments were also performed in rKSHV.219-infected EA.hy926 endothelial cells, which represent a biologically relevant KSHV infection model. To this end, latently infected rKSHV.219-Vero and -EA.hy926 cells were transiently transfected with V5-tagged Pim-1, -2 or -3 cDNAs or with an empty vector control. After 48 h, basal reactivation was induced using a low-titer recombinant baculovirus encoding RTA (BacK50) as described in Materials and Methods, and RFP expression was monitored 30 h later (Figure 1A and 1D). The basal reactivation induces low level of RFP expression (2.5% in rKSHV Vero, and 1.9% in rKSHV- EA.hy926) in the infected cells (negative controls), but is necessary for priming the cells for reactivation by transfected Pim-1 [25]. For positive controls, maximal viral reactivation was induced by treatment with high-titer RTA-encoding baculovirus (BacK50) and sodium butyrate (NaB). Interestingly, all Pim kinases induced RFP expression to at least 3-fold more as compared to the level obtained by basal reactivation (negative controls), with Pim-1 inducing the strongest (4- to 7-fold) increase in both cell lines. Reactivation by Pim kinases was stronger in Vero than in EA.hy926 cells, which is in accordance with the ability to obtain higher maximal reactivation efficiency in these cells by BacK50 and NaB (Figure S1A). Over-expression of irrelevant kinases CDK7 or LKB1, did not induce any significant reactivation over the negative control (Figure S1A).

To study the requirement of kinase activity for reactivation, we used mutant cDNAs of the Pim kinases from the kinome library [25]. The ATP binding site of the kinases was disrupted by a single point mutation (K67M in Pim-1, K61M in Pim-2, and K69M in Pim-3), rendering the kinases inactive. After transfection of the individual kinase-deficient (KD) mutants, reactivation levels were close to that of the negative controls (Figure 1A and 1D), suggesting that kinase activity was required for reactivation.

To analyze whether expression of Pim kinases was sufficient to trigger the complete lytic replication cascade, we analyzed the production of progeny virions to the supernatant of the transfected rKSHV-Vero or -EA.hy926 cells by monitoring GFP expression in naïve human osteosarcoma (U2OS) cells infected with the supernatants as described in Materials and Methods (Figure 1B and 1E). The U2OS cells were chosen as target cells due to their good susceptibility to KSHV infection, and suitable morphology for automated microcopy (our unpublished results). All wild-type (wt) Pim kinases induced a 2- to 5-fold increase in GFP in the U2OS cells infected with supernatants from the rKSHV-Vero or -EA.hy926 cell lines when compared to the negative controls, suggesting that the expression of Pim kinases induced completion of the full lytic cascade. Importantly, production of infectious virions was also dependent on kinase activity as expression of the V5-tagged kinase-deficient Pim kinases, the Pim-1KD, -2KD and -3KD, in both cell lines led to a clear reduction (maximally 10-fold) in GFP expression as compared to their wt counterparts. Over-expression of irrelevant kinases CDK7 or LKB1, did not induce increase in GFP expression over the negative control in U2OS target cells (Figure S1B).

To further confirm activation of the full lytic program, expression of the late lytic protein K8.1 was analyzed in rKSHV-Vero and -EA.hy926 cell lines transfected with the wt and KD Pim kinases. In accordance with the analysis for GFP expression above, K8.1 expression was induced in cells transfected with wt Pim kinases, but not in cells transfected with the mutants (Figure 1C and 1F). Equal expression of individual wt and mutant Pim kinases was confirmed by Western blotting using V5-antibodies (Figure 1C and 1F). Although Pim-3 wt and KD were expressed at lower levels in rKSHV-Vero cells than the other Pim kinases, induction of K8.1 by wt Pim-3 was equally efficient.

### Pim-1 and -3 are involved in KSHV reactivation

**Figure 2:**
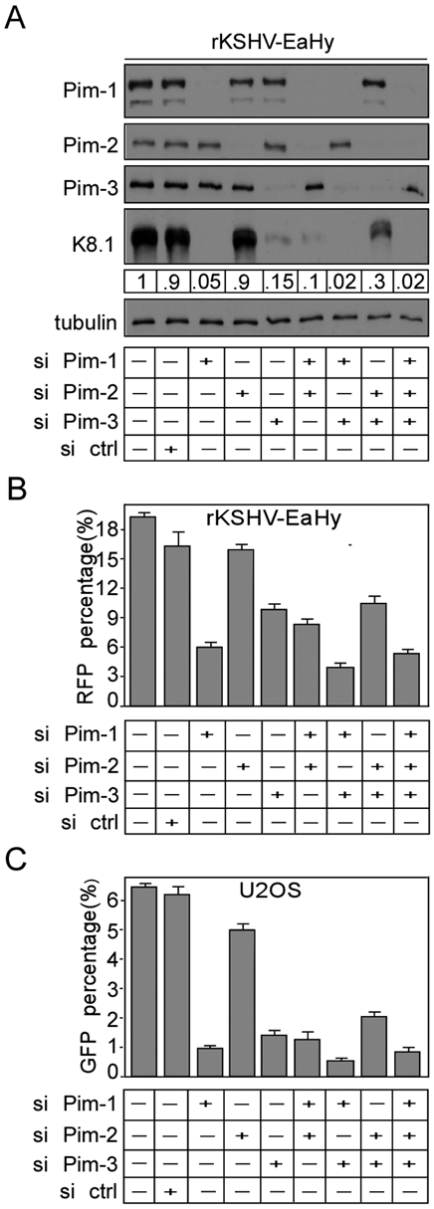
Pim-1 and -3 are required for KSHV reactivation. (A) rKSHV-EA.hy926 cells (rKSHV-Ea.hy) were transfected with siRNA oligonucleotides specific for Pim-1, -2, or -3, or with control siRNA (si ctrl), and subjected to maximal reactivation (RTA+NaB) 48 h after transfection. After another 48 h, whole cell extracts were collected and analyzed by Western blotting with anti-Pim-1, -2, -3, and -K8.1 antibodies. Quantification of K8.1 protein level is shown below the panel. Anti-tubulin served as a loading control. (B) Cells were treated as in (A), and 30 h after maximal reactivation, the cells were analyzed for RFP expression. 72 h after reactivation, supernatants were collected and used to infect naive U2OS cells. (C) 72 h after infection, the U2OS cells were analyzed for GFP expression (a measure of production of infectious virions). Values are means of two independent experiments ±SD.

To rule out the possibility that viral reactivation by Pim kinases was only observed as a result of over-expression, we silenced expression of all Pim kinases individually by RNA interference to analyze their requirement for viral reactivation in KSHV-infected cells. To this end, rKSHV-EA.hy926 cells were transfected with siRNA oligonucleotides specific for Pim-1, -2 or -3, or with a non-targeting control siRNA. The cells were subjected to maximal reactivation 48 h after transfection. After another 48 h, lysates were collected and efficient depletion of Pim-1 (85%), Pim-2 (90%) and Pim-3 (80%) protein expression was confirmed by immunoblotting (Figure 2A). The effect of silencing on viral reactivation was examined by immunoblotting with K8.1 (Figure 2A) as well as by measuring the induction of RFP, and expression of GFP on naïve U2OS cells as described in Figure 1 (Figure 2B). Importantly, depletion of Pim-1 and -3 expression almost completely inhibited induction of the late lytic marker K8.1 in rKSHV-EA.hy926 cells while Pim-2 and the control siRNAs had no effect (Figure 2A). Consistent with K8.1 expression, a significant reduction in the expression of RFP (2.6-fold and 1.6-fold, respectively) in rKSHV-EA.hy926 cells and GFP (6.5-fold and 4-fold, respectively) on U2OS cells was observed (Figure 2B) in the Pim-1 and Pim-3 depleted samples. However, Pim-2 silencing had very little effect on KSHV reactivation, suggesting that endogenous Pim-2 is not required for the induction of lytic replication or that the higher expression levels of the other two Pim family members mask the effects of the less abundantly expressed Pim-2 protein. Simultaneous knockdown of Pim-1 and Pim-3 had a synergistic effect on viral reactivation as observed by a greater reduction in RFP, GFP and K8.1 expression as compared to samples silenced with individual siRNAs (4.5-fold, 14-fold and 45-fold respectively). Silencing of an irrelevant kinase LKB1, however, had no effect on the expression of RFP in the rKSHV-EA.hy926 cells or GFP on U2OS cells (Figure S1C). Taken together, these results suggest that expression of Pim-1 and -3 is necessary for KSHV lytic reactivation under the reactivation conditions used here.

### Pim-1 and -3 are up-regulated upon chemical induction of the lytic phase and are required for KSHV reactivation in PEL cells

**Figure 3:**
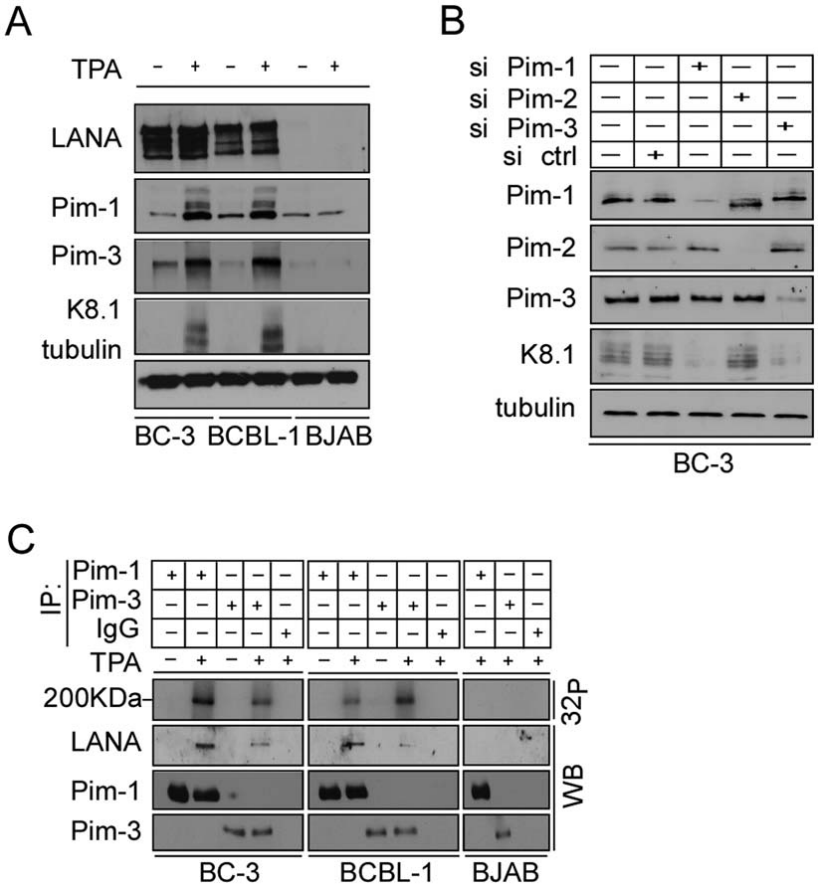
Pim-1 and -3 interact with and phosphorylate LANA upon reactivation of PEL cells. (A) BCBL-1, BC-3, as well as a KSHV–negative control cell line BJAB, were treated with TPA (+) or solvent (DMSO; −) for 48 h. Whole cell extracts were immunoblotted with anti-Pim-1, -3, and -K8.1 antibodies. Anti-tubulin served as a loading control. (B) BC-3 cells were transfected with siRNAs specific for Pim-1, -2, -3, or control siRNA (si ctrl), and treated with TPA 48 h after transfection. After another 48 h, whole cell extracts were collected and analyzed by Western blotting with anti-Pim-1, -2, -3, and -K8.1 antibodies. Anti-tubulin served as a loading control. (C) Whole cell extracts from (A) were immunoprecipitated with anti-Pim-1 and -3 or control IgG antibodies and subjected to an *in vitro* kinase assay towards co-precipitated proteins. The samples were resolved by SDS-PAGE (8%) followed by autoradiography. The kinase filter was immunoblotted with anti-LANA, -Pim-1, and -3 antibodies.

To gain more insight into the biological relevance of Pim kinase-mediated KSHV reactivation, we next analyzed the expression levels of endogenous Pim-1, -2 and -3 upon chemical reactivation of naturally KSHV-infected PEL cells. To this end, BC-3 and BCBL-1 PEL cells were first synchronized to S phase by serum starvation for 24 h, then cultured in the presence of serum for 16 h, followed by treatment with 20 ng/ml TPA for 48 h. efficient reactivation was confirmed by immunoblotting for the late lytic marker K8.1. Intriguingly, expression of Pim-1 and -3 was clearly up-regulated (2- 3-fold) in reactivated PEL cells, but not in latent or KSHV-negative control BJAB cells (Figure 3A), while the expression of Pim-2 remained unchanged (Figure S2). Moreover, silencing of Pim-1 or -3 expression by RNA interference in the TPA-reactivated BC-3 cells remarkably inhibited induction of the late lytic marker K8.1 (Figure 3B), indicating that Pim-1 and -3 are required for the reactivation. In accordance with the findings above, depletion of Pim-2 by siRNA had no effect on induction of K8.1 (Figure 3B). Interestingly, depletion of Pim-2 expression seemed to change the mobility of the Pim-1 band in the Western blot, suggesting a post-translational modification on the protein.

### Pim-1 and -3 interact with and phosphorylate LANA upon reactivation

A recent study by Bajaj et al [35] reported that upon co-transfection of Pim-1 and LANA, Pim-1 binds to the C-terminus of LANA and phosphorylates the protein *in vitro*. This prompted us to investigate if Pim-1 and -3 would phosphorylate LANA in PEL cells. To this end, whole cell extracts of untreated and TPA-treated cells described in Figure 3A were immunoprecipitated with anti-Pim-1, -Pim-3, or control IgG antibodies and subjected to an *in vitro* kinase assay on co-precipitated proteins followed by SDS-PAGE and autoradiography. Interestingly, LANA associated with both Pim-1 and -3 only in the reactivated cells, but not in latent cells. The detected interaction between LANA and Pim-1 or -3 in reactivated cells could, however, be simply due to the 2- to 3-fold increase in the levels of Pim-1 and -3 upon TPA treatment, while there might not be enough of these kinases present in the untreated, uninduced PEL cells to detect the interaction. To address this we immunoprecipitated Pim-1 from untreated BC-3 cell extracts with two (1200 µg) or three times (1800 µg) higher concentration of total protein as compared to Figure 3C, or from TPA-treated BC-3 extracts with 600 µg or 1200 µg of protein, and analysed the associated LANA by immunoblotting. As expected, increasing the protein concentration of the TPA-treated extract resulted in approximately twofold increase in the amount of co-precipitated LANA, while no association of LANA was observed in the Pim-1 immunoprecipitates from the more concentrated, untreated BC-3 extracts (Figure S3). This suggests that the interaction between Pim-1 and LANA is due to reactivation and not simply to an increase in Pim-1 protein levels.

Importantly, an approximately 200 kDa phosphorylated band was detected in the autoradiograph from the kinase assay of TPA-reactivated BC-3 cells where LANA was observed to co-precipitate with Pim-1 and -3 (Figure 3C, ^32^P, top panel). This band co-migrated with the lowest isoform of LANA in subsequent immunoblotting of the kinase filter (Figure 3C, LANA panel), suggesting that LANA was phosphorylated by Pim-1 and -3 in the reactivated cells. Similar results were obtained in PEL cell lines reactivated with NaB (data not shown). We did not observe co-precipitation or phosphorylation of LANA in the Pim-2 immunoprecipitates either in latent or reactivated PEL cells (data not shown). These results suggest that LANA is a substrate for Pim-1 and -3 kinases in reactivated PEL cells *in vivo*.

### LANA is phosphorylated by Pim-1 and Pim-3 at the N-terminus on serines 205 and 206

**Figure 4:**
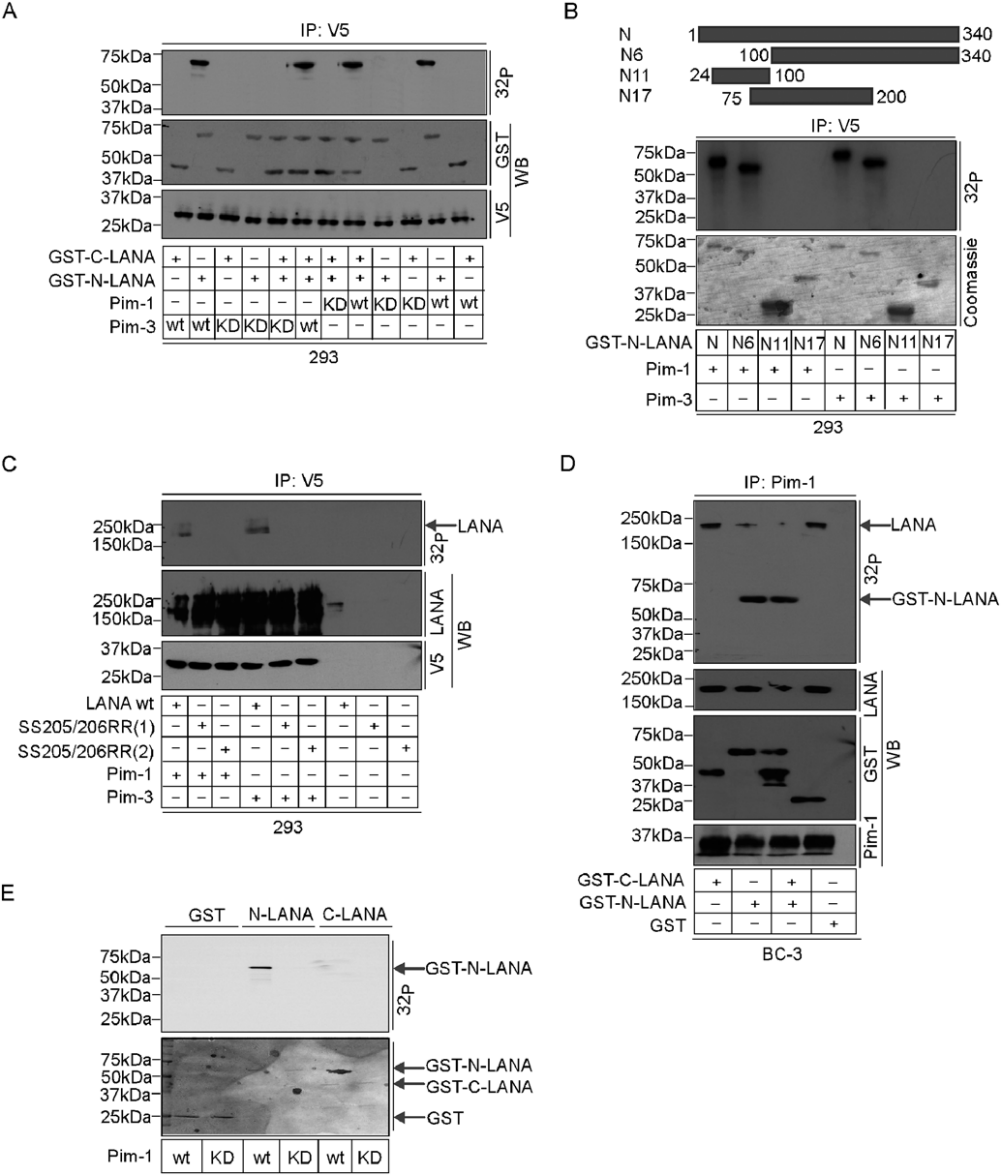
LANA is phosphorylated by Pim-1 and Pim-3 at the N-terminus on serines 205 and 206. (A) 293 cells were transfected with V5-tagged expression constructs for either wt or kinase-deficient (KD) Pim-1 or -3. After 48 h, cell extracts were immunoprecipitated by anti-V5 antibodies and subjected for in vitro kinase assay on GST-C-LANA or GST-N-LANA. The samples were resolved by SDS-PAGE (8%), and followed by autoradiography. The kinase filter was blotted with anti-GST antibody and anti-V5 antibodies. (B) The truncated fragments of the LANA N-terminus are illustrated in the upper part of the panel. 293 cells were transfected with V5-tagged expression constructs for either Pim-1 or -3. After 48 h, the in vitro kinase assay was performed essentially as described in (A) on GST-fusions of N-terminal truncations of LANA as indicated. Coomassie staining was used to detect the substrates. (C) 293 cells were transfected with wt (LANA wt) or two different clones of the SS205/206RR mutant of LANA (SS205/206RR (1), and SS205/206RR (2)) with and without V5-tagged expression constructs for Pim-1 or -3 as indicated. After 48 h, cell extracts were collected and subjected to an *in vitro* kinase assay essentially as described in (A) on co-precipitated proteins. The samples were resolved by SDS-PAGE (8%) followed by autoradiography. The kinase filter was immunoblotted with anti–LANA, and anti-V5 antibodies to ensure the efficiency of immunoprecipitation. An arrow indicates phosphorylated LANA band on the right. (D) BC-3 cells were treated with TPA for 48 h. Whole cell extracts were incubated with GST-N-LANA and GST-C-LANA alone or in combination for 6 hrs and subjected to immunoprecipitation with anti-Pim-1 antibody, followed by *in vitro* kinase assay on co-precipitated proteins. GST alone (GST) was used as a negative control. The kinase filter was immunoblotted with anti–LANA, –GST, and –Pim-1 antibodies. The arrows indicate phosphorylated LANA and GST-N-LANA on the right. (E) Recombinant GST-fusion proteins (GST, GST-N-LANA, or GST-C-LANA) were incubated with purified wild-type (wt) or kinase-dead (KD) Pim-1 proteins and subjected to an in vitro kinase assay. Samples were resolved by SDS-PAGE and autoradiographed. The substrates indicated on the right were visualized by Coomassie staining.

The recent work by Bajaj et al [35] identified serines 205 and 206 as Pim-1 phosphorylation sites within the amino-terminal domain of LANA. To confirm this and to study whether the N-terminus of LANA is targeted also by Pim-3, we transfected the V5-tagged Pim-1 and -3 wt and KD expression vectors into 293 cells, and performed immunoprecipitation with anti-V5 antibodies followed by an *in vitro* kinase assays using recombinant N-terminal (GST-N-LANA) and C-terminal (GST-C-LANA) LANA as substrates. In accordance with the finding of Bajaj et al [35] with Pim-1, both Pim-1 and -3 phosphorylated GST-N-LANA while no phosphorylation was observed on GST-C-LANA or with Pim-1KD or Pim-3KD (Figure 4A). To further map the site of Pim-1 and -3 phosphorylation on the LANA N-terminus, we prepared truncated versions of the GST-N-LANA as described in the Materials and methods, and used them as substrates in the *in vitro* kinase assay as described above. The results demonstrated that the critical residues for phosphorylation by Pim-1 and -3 were located between the amino acids 200 and 340 (Figure 4B). To define the specific phosphorylation sites on LANA, we prepared a site-specific LANA phosphomutant identical to the one described earlier by Bajaj et al [35] where the two critical serines at positions 205 and 206 were mutated into arginines (SS205/206RR). The wt and two different clones of the SS205/206RR mutant of LANA (SS205/206RR (1) and SS205/206RR (2)) were then transfected in the presence or absence of V5-tagged Pim-1 or -3, and cell extracts were subjected to immunoprecipitation with anti-V5 antibodies. When *in vitro* kinase assays were performed on the co-precipitated LANA proteins, we observed that wt LANA was phosphorylated by both Pim-1 and -3, while the SS205/206RR mutant of LANA failed to be phosphorylated by neither of them (Figure 4C). Neither of the LANA proteins was phosphorylated in the absence of transfected Pim kinases. These data suggest that serines 205 and 206 on the LANA N-terminus are specifically phosphorylated by Pim-1 and Pim-3.

To provide additional evidence that the 200-kDa band co-precipitated and phosphorylated by Pim-1 from TPA-induced BC-3 cells (Figure 3C) is indeed LANA we performed a competition experiment where GST-N-LANA and/or GST-C-LANA were added as competing substrates into the cell extract prior to Pim-1 immunoprecipitation from BC-3 cells that had been treated with TPA for 48 h. The immunoprecipitates were then subjected to an *in vitro* kinase assay on co-precipitated proteins. Purified GST was used as a negative control. As shown in Figure 4D, the phosphorylation signal on the 200- kDa band was dramatically reduced in samples containing GST-N-LANA while no effect was observed with GST-C-LANA or GST alone. This suggests that the phosphorylated 200-kDa band is indeed LANA. To obtain further support that the kinase responsible for the phosphorylation of LANA is Pim-1, instead of another kinase co-precipitating with Pim-1, we performed an *in vitro* kinase assay with purified wt and KD Pim-1 proteins and used GST-N-LANA, GST-C-LANA or GST as substrates. Phosphorylation was detected on GST-N-LANA, but not on GST-C-LANA or GST, only in the presence of the purified wt Pim-1 kinase (Figure 4E), demonstrating direct phosphorylation of the LANA N-terminus by Pim-1. Phosphorylation of GST-N-LANA on both Ser 205 and 206 was further confirmed by mass spectrometry (data not shown). These data confirm that serines 205 and 206 on the LANA N-terminus are the specific residues phosphorylated by Pim-1.

### Phosphorylation of LANA by Pim-1 and -3 counteracts the ability of LANA to inhibit transcription from the terminal repeat region

**Figure 5:**
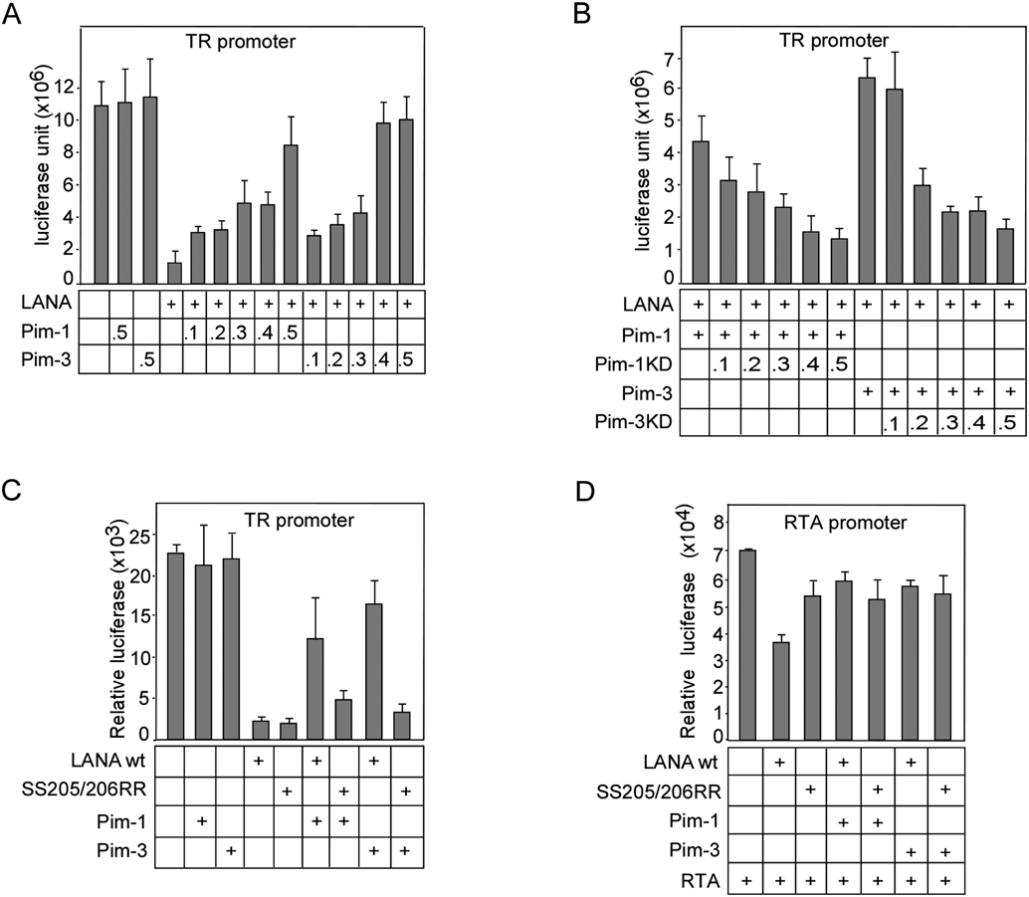
Phosphorylation of LANA by Pim-1 and -3 antagonizes LANA–mediated transcription inhibition. (A) The reporter plasmid pGL3-7XTR (0.1 µg) was co-transfected with expression vectors for LANA (0.5 µg) with and without increasing concentrations of V5-tagged vectors for Pim-1 or -3 (indicated below the graph) into 293 cells. The total transfected DNA concentrations (µg) were kept constant by including an empty control vector. 48 h after transfection, cell extracts were collected for the luciferase assay. (B) Increasing amounts of V5-tagged Pim-1KD or -3KD expression vectors were co-transfected with constant amount of pGL3-7XTR (0.1 µg), LANA (0.5 µg), and V5-Pim-1 or -3 (0.5 µg each) wt vectors into 293 cells. The DNA concentrations (µg) for Pim-1KD and -3KD cDNAs used are indicated below the graph, while the total DNA concentrations (µg) were kept constant by including an empty control vector. Cell extracts were analyzed as in (A). (C) The pGL3-7XTR (0.1 µg), V5-tagged Pim-1 or -3 (0.5 µg each) wt vectors were co-transfected with 0.5 µg of wt LANA or SS205/206RR mutant of LANA into 293 cells. At 48 h after transfection, dual luciferase assays were performed with extracts of transfected cells. An empty pcDNA3 was used as a filler plasmid to keep the total DNA constant in each transfection. Values represent an average of two independent experiments ±SD. (D) The reporter plasmid pGL2-ORF50p (0.1 µg), as well as expression vectors for RTA (0.5 µg) and wt LANA or SS205/206RR mutant of LANA (0.5 µg) were co-transfected with V5-tagged cDNAs for Pim-1 or -3 (indicated below the graph) into 293 cells. The transfected DNA concentrations (µg) were normalized by including an empty control plasmid. 48 h after transfection, cell extracts were collected for the luciferase assay. Values represent an average of two independent experiments ±SD.

A previous study has reported that the C-terminus of LANA binds specifically to sequences within the terminal repeat (TR) regions of the KSHV genome [36]. Furthermore, by using a reporter construct consisting of multimerized TR repeats linked to a heterologous promoter for luciferase gene expression (pGL3-7xTR), this and other studies [36] showed that transcription was suppressed up to 10-fold in the presence of LANA. We next addressed the effect of Pim-1 or -3 over-expression on the LANA-mediated repression of the pGL3-7xTR reporter. To this end, increasing amounts of Pim-1 or -3 were co-transfected with LANA and pGL3-7xTR, and the cells were subjected to luciferase reporter assays 48 h after transfection. Equal expression of LANA was confirmed by immunoblotting (data not shown). Ectopic expression of increasing amounts of Pim-1 or -3 counteracted LANA-mediated transcriptional repression of the TR-containing reporters (Figure 5A). Expression of Pim-1 or -3 did not influence transcription from TR-containing reporters in the absence of LANA, suggesting that Pim-1 or -3 does not directly enhance transcription from the reporter.

To test the requirement of kinase activity for the de-repression, we transfected increasing amounts of Pim-1KD and -3KD together with constant levels of LANA, wt Pim-1 and -3, as well as pGL3-7xTR. The samples were subjected to the luciferase reporter assays 48 h later. Expression of increasing amounts of Pim-1KD and -3KD neutralized the effect of Pim-1 and Pim-3 on LANA-mediated repression and resulted in a dose-dependent suppression of transcription from the TR-containing reporters (Figure 5B). To address whether phosphorylation of LANA at SS205/206 was necessary for the de-repression, we co-transfected cells with pGL3-7xTR, wt or SS205/206RR mutant of LANA (SS205/206RR) with and without Pim-1 or Pim-3. The samples were subjected to luciferase reporter assays 48 h after transfection. Expression of the LANA phosphosite mutant without the Pim kinases induced similar repression of luciferase activity as the wt LANA, but co-expressed Pim-1 and -3 were unable to relieve this repression (Figure 5C). These results indicate that phosphorylation of LANA at serines 205 and 206 by Pim-1 or -3 is needed to counteract LANA-mediated suppression of transcription from the TR.

### Pim-1 and -3 counteract the ability of LANA to inhibit RTA autoactivation

Induction of K8.1 upon Pim-1 and -3 over-expression or in response to TPA-induced reactivation suggests that RTA responsive lytic genes are also activated by these kinases in KSHV-infected cells. Since RTA is not located in the proximity of the TR region in the KSHV genome we wanted to investigate whether co-expression of Pim-1 or -3 could affect the ability of LANA to inhibit transcription from the RTA promoter. Previous studies have demonstrated that RTA can auto-activate its own viral promoter [37], and this was shown to be partially repressed by LANA [38]. We therefore addressed the ability of LANA to repress transcription of luciferase from a RTA-Luc reporter (pGL2-ORF50p) upon co-expression of RTA, wt Pim-1 or -3. In accordance with Lan et al [38] expression of LANA led to a partial repression of the RTA-Luc reporter activity. Moreover, Pim-1 and -3 wt were both able to de-repress the RTA-Luc activity (Figure 5D). Equal expression of LANA was confirmed by immunoblotting (data not shown). To address whether phosphorylation of LANA at SS205/206 was necessary for the de-repression, we co-transfected cells with pGL2-ORF50p, wt or SS205/206RR mutant of LANA (SS205/206RR) with and without Pim-1 or Pim-3. Expression of the LANA phosphosite mutant without the Pim kinases was able to repress the RTA-luciferase activity but to a lesser extent as the wt LANA. Yet, co-expressed Pim-1 and -3 were unable to relieve this repression (Figure 5D). Taken together, this suggests that expression of Pim-1 and -3 kinases can antagonize the ability of LANA to inhibit autoactivation of the RTA promoter.

### IFN-gamma increases Pim-1 levels and induces reactivation in PEL cells

**Figure 6:**
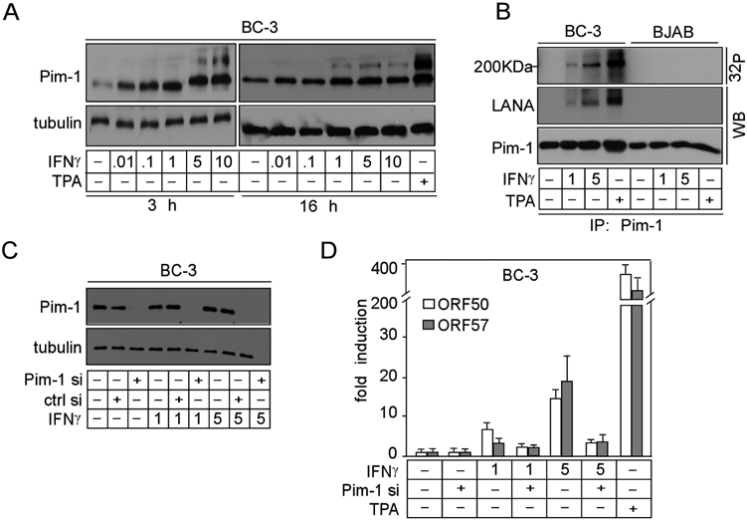
IFNγ induces reactivation via Pim-1. BC-3 cells were first synchronized to S (Pim-1 si)-phase and treated with increasing amount of IFN-γ for 3 or 16 h. TPA treatment was used as a positive control. (A) Cell extracts were analyzed for the expression of Pim-1 by Western blot, and (D) for expression of ORF50 and ORF57 lytic transcripts by qRT-PCR. (B) BC-3 and BJAB cells were treated with 1 or 5 U/ml of IFN-γ for 16 h, and whole cell extracts were immunoprecipitated with anti-Pim-1 antibodies and subjected to an *in vitro* kinase assay on co-precipitated proteins. The samples were resolved by SDS-PAGE (8%) followed by autoradiography. The kinase filter was immunoblotted with anti–LANA and anti–Pim-1 antibodies. Anti-tubulin served as a loading control. (C) BC-3 cells were transfected with siRNAs specific for Pim-1 (Pim-1 si) or control siRNA (ctrl si) or left untreated (−). 48 h after transfection, cells were treated for 16 h with IFN-γ as described above. Cell extracts were analyzed for the silencing of Pim-1 expression by Western blot with anti-Pim-1 antibodies, and (D) for expression of ORF50 and ORF57 lytic transcripts by qRT-PCR. Anti-tubulin served as a loading control in C.

To further assess the role of Pim kinases in KSHV reactivation upon physiological instead of chemical induction, we next analyzed the expression levels of endogenous Pim-1 in PEL cells upon cytokine stimulation. To this end, BC-3 cells were first synchronized as described in Figure 3 and treated with increasing amounts of interferon-γ (IFN-γ) for 3 or 16 h. Expression of Pim-1 was moderately up-regulated in BC-3 cells already 3 h after IFN-γ treatment (Figure 6A). In addition, a slower migrating form of Pim-1 appeared in samples treated with 5–10 IU/ml or 1–10 IU/ml for 3 h or 16 h, respectively, suggesting a post-translational modification on the protein. Next we examined the effects of IFN-γ on the expression of KSHV lytic genes by quantitative real-time PCR (qRT-PCR). At 16 h IFN-γ treatment with 0.1–10 IU/ml induced the expression of ORF50/RTA (immediate-early transcript) up to 16.5-fold and ORF57 (delayed-early transcript) up to 19-fold in a dose-dependent manner (Figure 6D), implying that cytokine treatment lead to expression of viral lytic genes. We also addressed if Pim-1 phosphorylated LANA in IFN-γ stimulated PEL cells. To this end, whole cell extracts of untreated, IFN-γ- or TPA-treated BC-3 (16 h stimulation) or control BJAB cells were immunoprecipitated with anti-Pim-1 antibodies and subjected to *in vitro* kinase assays on co-precipitated proteins followed by SDS-PAGE and autoradiography. In accordance with the induction of lytic gene expression, an approximately 200 kDa band co-migrating with LANA in subsequent immunoblotting was detected in the autoradiograph of Pim-1 immunoprecipitates after treatment of BC-3 cells with either IFN-γ or TPA (Figure 6B, ^32^P, top panel), further supporting the physiological role for Pim-1 in KSHV reactivation.

To study the requirement of Pim-1 for the IFN-γ induced KSHV reactivation, we silenced Pim-1 expression by RNA interference in BC-3 cells as described for Figure 3, and confirmed the depletion by Western blotting (Figure 6C). 48 h after the cells were treated with 1 or 5 U/ml of IFN-γ for 16 h, and viral reactivation was analysed by qRT-PCR for the expression of lytic genes ORF50 and ORF57. Silencing of Pim-1 expression led to a significant decrease in the expression of lytic genes (66% and 77% reduction for ORF50, and 38% and 81% reduction for ORF57 with 1 and 5 U/ml IFN-γ treatment, respectively) as compared to cells treated with the control siRNA (Figure 6D and not shown). Taken together, this data suggests that IFN-γ-induced KSHV reactivation occurs via activation of Pim-1.

## Discussion

**Figure 7:**
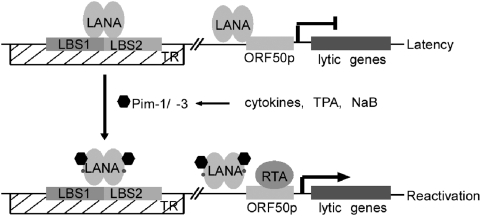
A model for Pim-1 and Pim-3 function in KSHV reactivation. In latency, binding of LANA to the LANA binding sites 1 and 2 (LBS 1 and LBS2) at the terminal repeat (TR) region represses transcription from the TR region. LANA can also repress transcription from the RTA promoter and inhibit RTA to autoactivate its own promoter. Upon stimulation by cytokines, TPA or NaB, Pim-1 and -3 are up-regulated, and they interact with and phosphorylate LANA. The phosphorylation by Pim-1 and -3 counteracts the LANA–mediated inhibition of transcription from the TR region and down-modulates autoactivation of the RTA-promoter, leading to viral reactivation.

In this report, we have identified two cellular kinases, Pim-1 and Pim-3, as critical regulators of KSHV viral reactivation. During reactivation, Pim-1 and -3 interact with and phosphorylate LANA in both *de novo* and naturally KSHV-infected cells. LANA can repress expression of a subset of lytic genes by specifically binding to the LANA binding sites 1 and 2 (LBS1 and LBS2) within the TR region of the KSHV genome [39],[40]. Here we demonstrate that phosphorylation by Pim-1 and -3 counteracts this LANA-mediated inhibition of transcription from the TR region. Besides inhibiting the TR-enhancer dependent transcription LANA can down-modulate the transcriptional activity of the RTA gene promoter as well as the ability of RTA to autoactivate its own promoter [38]. Our data shows that co-expression of Pim-1 and -3 with LANA was able to de-repress the LANA-induced partial inhibition of autoactivation of the RTA-promoter (Figure 5). However, this Pim kinase-mediated de-repression or the inhibitory effect of LANA phosphosite mutant was not as dramatic as those seen in the TR-enhancer dependent transcription assay. This variation could be due to different mechanisms behind the repression. While the LANA-induced inhibition of transcription from the TR region depends on direct binding of LANA to DNA at LBS1 and LBS2 [39],[40], the suppression of RTA autoactivation occurs through direct binding of LANA to RTA [38]. Taken together, our results suggest that phosphorylation of LANA by Pim kinases can modulate its ability to interact with the enhancer elements within the TR region and with the RTA protein itself, and thereby lead to viral reactivation (Figure 7).

Our KSHV-infected cell models manifest a very low level of spontaneous lytic replication. However, upon stimulation by low level of exogenous RTA (using a low-titer BacK50) and co-expression of Pim kinases, a relatively large population of cells undergoes reactivation. There are several possible reasons to explain why priming with exogenous RTA is needed to induce the Pim kinase-mediated lytic replication. One explanation is that as the latency program in these infected cell models is very tight, a threshold level of RTA might be required for the activation of viral lytic cascade by Pim. Alternatively, Pim kinases may also have an effect on RTA itself.

Bajaj et al [35] recently demonstrated that Pim-1 phosphorylates LANA at either one or both of the serines at positions 205 and 206. We identified Pim-3 as a novel kinase phosphorylating LANA in naturally infected PEL cells, and demonstrated that the phosphorylation takes place only upon viral reactivation. Our data confirms Ser 205/206 as the target sites for Pim-1 and demonstrate that these sites are phosphorylated also by Pim-3. Pim kinases are involved in partially overlapping survival pathways and are implicated in a wide variety of human diseases including cancer, inflammatory disorders, and ischemic diseases [26],[41]. Moreover, they have previously been linked to regulation of viral oncogenesis [31],[32],[35]. Our data extend these observations by suggesting a novel link between Pim kinases and KSHV, and establishes Pim-1 and -3 as key factors in KSHV reactivation.

Our findings provide compelling evidence that Pim-1 and -3, but not Pim-2, are essential for KSHV reactivation both in *de novo* infected endothelial cells and in the naturally infected PEL cells. Interestingly, we observe a surprising additive effect of Pim-1 and Pim-3 as depletion of one of them results in almost equal level of inhibition of reactivation. One possible explanation could be that the KSHV-infected cells require a threshold level of active Pim kinases to reactivate, and that endogenous levels of Pim-2 would be below the limit, and thus its depletion does not affect reactivation. When expression of Pim-1 or -3 is efficiently silenced, the total Pim kinase activity may fall below the required threshold, leading to inhibition of reactivation upon depletion of either of them.

A number of inflammatory and angiogenic cytokines including IL-6, tumor necrosis factor-α, IFN-γ, platelet-derived growth factor, oncostatin M and interleukin-1β are expressed in KS tumors [42]. Some of these cytokines have been suggested to modulate KSHV reactivation [43]. Intriguingly, expression of Pim-1 is induced by a number of cytokines, such as IFN-α, IFN-γ, IL-3, and IL-6 [26]. It is therefore plausible that the presence of a variety of cytokines in the microenvironment of KSHV-associated tumors may induce Pim kinase expression and/or activation and lead to viral reactivation in affected tissues. Our data demonstrating involvement of Pim-1 in IFN-γ treated PEL cells on activation of ORF50 and ORF57 lytic gene transcription supports a physiological role of Pim-1 in KSHV reactivation.

The switch from latency to lytic replication is an elementary decision in the viral life cycle and several lines of evidence support the importance of viral lytic replication in herpesviral tumorigenesis [7],[8]. A sustained, although low level of reactivation can contribute to progression of KS by not only producing new virions but also by contributing to other aspects of KS pathogenesis, such as the production of inflammatory and angiogenic cytokines. Hence, cellular signaling pathways operative during viral reactivation could represent potential novel targets for therapeutic intervention. Accordingly, inhibitors specific for either Pim-1 or -3 or a broad spectrum Pim inhibitor could be developed as antiviral agents, as inhibition of either Pim-1 or -3 results in a significant decrease in reactivation. These inhibitors could also be tested for their anti-tumor activity in PEL mouse models to explore a novel therapeutic strategy to treat KSHV-malignancies. As animals lacking all three Pim genes are viable with a mild phenotype [44], one could anticipate that small-molecule inhibitors targeting Pim-1 and -3 might be tolerated without significant side effects.

## Materials and Methods

### Cells

Vero cells latently infected with a recombinant GFP-expressing KSHV (rKSHV.219) were a kind gift from J. Vieira (Univ. of Washington). EA.hy926 cells infected with rKSHV.219 were prepared as described in [45]. The BC-3 and BCBL-1 cell lines were kindly provided by E. Cesarman (Cornell Medical College) and and the BJAB B-lymphoblastoid cell line was a gift from J. Salonen (Univ. of Tampere). BC-3, BCBL-1 and BJAB cells were cultured in a humidified 5% CO2 atmosphere at 37°C in RPMI 1640 medium supplemented with 10% fetal calf serum (FCS; Invitrogen, CA), 100 U/ml penicillin G, and 100 µg/ml streptomycin. 293 and U2OS human osteosarcoma cell lines (ATCC; Manassas, VA), rKSHV-Vero, and -EA.hy926 cells were cultured similarly, but with Dulbecco modified Eagle's medium (DMEM). In addition, 5 ug/ml or 1 ug/ml of puromycin was added to the medium for rKSHV-Vero or rKSHV-EA.hy926 cells, respectively.

### Plasmids

Wild-type and kinase-deficient (KD) versions of human Pim-1, Pim-2 and Pim-3 expression vectors were generated by PCR amplification and cloning into the pcDNA3.1/V5-HisC vector [25]. The GST-tagged Pim-1 and Pim-1KD constructs were generated by PCR subcloning into the pGEX-6P-1 vector (GE Healthcare). pGEX-2T plasmid (GE Healthcare) was used to produce GST in control samples. The serine to arginine (SS205/206RR) mutations on LANA were generated using pCDNA3.1-LANA [46] as a template by site-directed mutagenesis. The GST-tagged carboxy-terminal LANA (GST-C-LANA; amino acids (aa) 972-1162) and amino-terminal LANA (GST-N-LANA; aa 1–340 (N-LANA) [47] were expressed from the pGEX4T1-LANA construct. The full length and truncated versions of the N-terminal LANA (aa 1–340 (N), aa 100–340 (N6), aa 24–100 (N11), and aa 75–200 (N17)) were generated by PCR amplification from the pCDNA3-LANA and fused to GST expression cassette by ligation to pGEX-4T-1 (GE Healthcare). The luciferase reporter construct in the pGL2-ORF50p backbone containing the RTA promoter (3 kb sequence upstream of the RTA translation initiation codon) was prepared as described before [37] and the luciferase reporter containing seven terminal repeats (TR) of KSHV genome, pGL3-7xTR, was a kind gift from Dr. Rolf Renne (University of Florida) [36].

### Viral reactivation assay

Viral reactivation was addressed in Vero and EA.hy926 cells stably infected with recombinant KSHV (rKSHV.219) [33]. The rKSHV.219 is a double-reporter virus, which expresses the green fluorescent protein (GFP) from a constitutively active EF-1-promoter, and can be induced to express the red fluorescent protein (RFP) from the RTA-responsive KSHV lytic promoter for polyadenylated nuclear RNA (PAN) [34]. To address the effect of Pim kinases or their mutants on reactivation, rKSHV.219-Vero and -EA.hy926 cells were seeded on 6- or 24-well dishes and transiently transfected with the empty vector pcDNA3.1 or vectors expressing wt or kinase-dead Pim family members, as described previously [25]. In brief, 2×10^5^ rKSHV.219-Vero and -EA.hy926 cells per well were plated on a 6-well plate. On the following day, 1 µg aliquots of DNA samples were mixed with serum-free medium containing 3 µl FuGENE HD (Roche, Nutley, NJ) according to the manufacturer's protocol. One day after transfection, the media was changed into fresh DMEM media. To monitor transfection efficiency, pcDNA/DsRed plasmid was transfected into duplicate wells and the number of dsRed-positive cells was determined by fluorescence microscopy. The typical efficiency of transfection was between 15–20%. After 48 h, cells were treated for 2 h with a low-titer baculovirus (BacK50) expressing the KSHV lytic activator ORF 50/RTA (a kind gift from Dr. J. Vieira; Univ. of Washington). This triggered basal reactivation but led to a low level of RFP induction (2.5% in rKSHV-Vero, and 1.9% in rKSHV- EA.hy926) over the non-treated control (0.4% in rKSHV-Vero, and 0.05% in rKSHV-EA.hy926). Cells transfected with the control vector and treated as above served as negative controls, while positive controls were prepared by treating these cells for 2 h with a high-titer BacK50, followed by replacement with media containing 1.25 mM sodium butyrate (NaB, Sigma) to induce maximal reactivation (25% in rKSHV.219-Vero and 13% in rKSHV.219-EA.hy926 cells). 30 h after baculovirus infection, cells were fixed with 4% paraformaldehyde (PFA) and red fluorescent protein (RFP) expression was monitored using an automated high-content fluorescence microscope Arrayscan 4.5 (Cellomics). To measure completion of the lytic cascade and production of progeny virions, naïve target cells (U2OS) were infected 72 h later with 500 µl of the supernatant from transfected/reactivated cells in the presence of 8 ug/ml polybrene to enhance the infectivity. Plates were spin-transduced by centrifugation at 1,050 g for 30 minutes at room temperature and returned to 37°C, 5% CO2 for 2 h, after which the supernatant was replaced with complete media. 72 h after infection, target U2OS cells were fixed by 4% PFA and green fluorescent protein (GFP) intensity was analyzed by Cellomics Arrayscan 4.5.

### Antibodies, reagents, and siRNA transfections

The following antibodies were used for Western blotting or immunoprecipitation: anti–Pim-1 (12H8, Santa Cruz, CA), anti–Pim-2 (Atlas, Stockholm, Sweden), anti-Pim-3 (C-term, Abgent, San Diego, CA), anti-LANA, anti-K8.1 (ABI Biotechnologies, Columbia, MD), anti-V5-epitope (Invitrogen, Carlsbad, CA), anti-tubulin (5H1) (BD Biosciences, San Jose, CA). Protein-G Sepharose, Bisbenzimide Hoechst 33342, TPA and NaB were obtained from Sigma-Aldrich Chemical (St Louis, MO). IFN-γ was kindly provided by I. Julkunen (National Public Health Institute, Helsinki). The siRNA oligonucleotides targeting Pim-1, -2, -3 (ON-TARGET*plus* siRNAs) and a non-target control siRNA were obtained from Dharmacon (Chicago, IL). The rKSHV-EA.hy926 cells were seeded on 6- or 24-well dishes and transfected with 100 ng (per well on a 6-well dish) or 50 ng (per well on a 24-well dish) of the siRNA oligonucleotides targeting Pim-1, -2, -3 or a control non-targeting siRNA using DharmaFECT 1 (Dharmacon, Chicago, IL) according to manufacturer's instructions. 2×10^6^ BC-3 cells per well were seeded on a 6-well plate and transfected with 300 ng siRNA oligonucleotides per well using the Oligofectamine reagent (Invitrogen, Carlsbad, CA) according to manufacturer's instructions.

### Purification of the GST proteins

GST-proteins were produced in E. coli BL21 (GST-Pim-1 and -Pim-1KD) or DH5α cells (the GST-LANA proteins). Briefly, bacteria were grown at 30°C for the GST-Pim-1, -Pim-1KD, and for all the GST-N-LANA forms (full length and truncations), or at 25°C for the GST-C-LANA. The proteins were induced with 200 nM IPTG for the GST-Pim-1, -Pim-1KD, 100 µM for all the GST-N-LANA forms for 6 h, or with 100 µM for the GST-C-LANA for 16 h before the cells were harvested. The bacteria were lysed in PBS containing 0.5% Triton X-100 and protease inhibitors (Complete Mini EDTA- free, Roche) and sonicated. Solubilized GST-proteins were bound to glutathione sepharose beads (GE Healthcare) and eluted with buffer containing 30 mM glutathione in 75 mM Tris, pH 8.0. To cleave off the GST-tag from the Pim-kinases PreScission protease was used according to the manufacturer's instructions (GE Heatlhcare).

### Western blotting and quantitative image analysis

Cells were lysed in ELB lysis buffer (150 mM NaCl; 50 mM HEPES, pH 7.4; 0.1% Igepal; 5 mM EDTA; 2 mM DTT; 1 mM phenylmethylsulfonyl fluoride [PMSF]; 2 µg/ml leupeptin; 2 µg/ml pepstatin; and 1.5 µg/ml aprotinin) and lysates were then clarified by centrifugation at 20 800 *g* for 15 minutes at 4°C. Western blotting analysis was carried out as described previously [47],[48]. Quantitative protein analysis was performed using Single Dimensional Electrophoretic Gel Analysis program from the ImageJ software package, version 1.38 (NIH).

### Immunoprecipitation and kinase assay

To induce KSHV lytic replication, BC-3, BCBL-1 and control BJAB cells were treated with either 20 ng/ml TPA or 2 mM NaB for 48 h prior to lysis into the ELB lysis buffer (150 mM NaCl; 50 mM HEPES, pH 7.4; 0.1% Igepal; 5 mM EDTA; 2 mM DTT; 1 mM phenylmethylsulfonyl fluoride [PMSF]; 2 µg/ml leupeptin; 2 µg/ml pepstatin; and 1.5 µg/ml aprotinin). 600 µg of the whole-cell extracts were incubated with anti-Pim-1 or -3 antibodies for 2 h at +4°C. Immunocomplexes were coupled to protein-A or G Sepharose beads for an additional 1.5 hour at 4°C and washed 3 times with the lysis buffer followed by one wash with the kinase buffer (20 mM Tris-HCl, pH 7.5; 50 mM KCl; 7.5 mM MgCl_2_; 1 mM DTT; 25 mM ß-glycerophosphate; leupeptin 2 µg/ml; pepstatin 2 µg/ml; and aprotinin 1.5 µg/ml). Complexes bound to protein-A/G beads were either directly subjected to a kinase reaction towards co-precipitated proteins or were supplemented with exogenous GST-fusion proteins as substrates. For *in vitro* kinase assays, wild-type and kinase-dead Pim-1 proteins produced in bacteria as GST-fusion proteins were purified and cleaved by Precission protease (GE Healthcare). Kinase reactions were performed in the presence of 2 µCi (0.074 MBq) of [^32^P] adenosine triphosphate (ATP) for 30 minutes at 30°C and stopped by boiling in Laemmli buffer for 10 min. Phosphorylated proteins were resolved in 8% SDS-PAGE, transferred onto nitrocellulose membranes (Schleicher and Schuell, Dassel, Germany) and analysed by autoradiography and Western blotting when needed. Equal loading of proteins was confirmed with Colloidal Coomassie staining or Ponceau.

### Reporter assays

The luciferase reporter plasmids were transfected with FuGENE HD (Roche, Nutley, NJ) into HEK-293 cells according to the manufacturer's protocol. All transfections were performed with equal amounts of DNA by normalization with empty vector. The pRL-CMV plasmid that constitutively expresses Renilla luciferase was included as an internal control. Relative luciferase activities were calculated by dividing the normalized firefly luciferase activity of each reporter by that of pGL3 or pGL2 plasmid in transfected cells. 24 h or 48 h after the transfection, cells were harvested, washed in PBS and lysed into the cell lysis buffer provided by the manufacturer of the Dual-Luciferase Reporter Assay System kit (Promega). 100 µl of the cell lysate was used for the reporter assay using a DCR-1 luminometer (DIGENE, Gaithersburg, MD). An aliquot of the cell lysate was used for Western blotting to ensure equal expression of the transfected cDNAs.

### Quantitative real-time PCR

Total RNA was prepared by using the RNAeasy Mini-kit with removal of genomic DNA (Qiagen, Valencia, CA). Reverse Transcription (RT) was performed with TaqMan RT Reagents kit (Roche Diagnostics, Indianapolis, IN) according to manufacturer's protocol. Quantitative real-time PCR was performed with SYBRGreen chemistry, PCR conditions and sets of primers for ORF50, ORF57 and human beta-actin were essentially as described previously [48].

### Accession numbers

The Genbank (http://www.ncbi.nlm.nih.gov/Genbank/) accession numbers for the genes and gene products discussed in this paper are *Pim-1* (NM_002648), *Pim-2* (NM_006875), *Pim-3* (NM_001001852), *LANA* (AF305694), *TR* (U75700), *RTA* (4961526), *PAN* (4961489), *CDK7* (1022), *LKB1* (6794).

## Supporting Information

Figure S1 Overexpression of irrelevant kinases does not lead to KSHV reactivation. (A) Vero or EA.hy926 cells latently infected with rKSHV were transfected with expression vectors of CDK7 or LKB1 kinase. Basal reactivation of cells was induced by infection with RTA baculovirus 48 h after transfection (negative control), and NaB and RTA baculovirus were used to induce maximal reactivation (positive control). 30 h later, the cells were analyzed for RFP expression. (B) 96 h after adding the RTA baculovirus, the supernatants from rKSHV-Vero or rKSHV-EA.hy926 were collected and used to infect U2OS target cells. 72 h after infection, the U2OS cells were analyzed for GFP expression. (C) rKSHV- EA.hy926 cells were transfected with siRNA specific for LKB1, a control siRNA (si ctrl), or left untreated (mock), and subjected to maximal reactivation (RTA+NaB) 48 h after transfection. 30 h after reactivation, the cells were analyzed for RFP expression (three bars on the left). 72 h after reactivation, supernatants were collected and used to infect näive U2OS cells. 72 h after infection, the U2OS cells were analyzed for GFP expression (three bars on the right). Values are means of two independent experiments ±SD.
(0.10 MB DOC)

Figure S2 Pim-2 expression levels do not increase upon TPA treatment. BC-3, BCBL-1 as well as a KSHV-negative control cell line BJAB were treated with TPA (+) or solvent (DMSO; −) for 48 h. Whole cell extracts were immunoblotted with anti-Pim-2 antibody. Anti-tubulin served as a loading control.
(0.05 MB DOC)

Figure S3 Interaction of LANA and Pim-1 occurs upon induction of viral reactivation. BC-3 cells were treated with TPA (+) or solvent (DMSO; −) for 48 h. The indicated amounts of cell extracts were immunoprecipitated with anti-Pim-1 antibody and subjected to an *in vitro* kinase assay towards co-precipitated proteins. Samples were resolved by SDS-PAGE (8%) followed by autoradiography. The kinase filter was immunoblotted with anti-LANA and -Pim-1 antibodies. The inputs (10%) are shown on the right.
(0.15 MB DOC)
