# Supplementary material for: KSHV Reactivation from Latency Requires Pim-1 and Pim-3 Kinases to Inactivate the Latency-Associated Nuclear Antigen LANA
Source: PLoS Pathog. 2009 Mar 6;5(3):e1000324. doi: 10.1371/journal.ppat.1000324 (PMC2648312; doi:10.1371/journal.ppat.1000324)
Supplement: Figure S1 — Overexpression of irrelevant kinases does not lead to KSHV reactivation. (A) Vero or EA.hy926 cells latently infected with rKSHV were transfected with expression vectors of CDK7 or LKB1 kinase. Basal reactivation of cells was induced by infection with RTA baculovirus 48 h after transfection (negative control), and NaB and RTA baculovirus were used to induce maximal reactivation (positive control). 30 h later, the cells were analyzed for RFP expression. (B) 96 h after adding the RTA baculovirus, the supernatants from rKSHV-Vero or rKSHV-EA.hy926 were collected and used to infect U2OS target cells. 72 h after infection, the U2OS cells were analyzed for GFP expression. (C) rKSHV- EA.hy926 cells were transfected with siRNA specific for LKB1, a control siRNA (si ctrl), or left untreated (mock), and subjected to maximal reactivation (RTA+NaB) 48 h after transfection. 30 h after reactivation, the cells were analyzed for RFP expression (three bars on the left). 72 h after reactivation, supernatants were collected and used to infect näive U2OS cells. 72 h after infection, the U2OS cells were analyzed for GFP expression (three bars on the right). Values are means of two independent experiments ±SD. (0.10 MB DOC) [file ppat.1000324.s001.doc]

**Figure S1. Overexpression of irrelevant kinases does not lead to KSHV reactivation.** **(**A) Vero or EA.hy926 cells latently infected with rKSHV were transfected with expression vectors of CDK7 or LKB1 kinase. Basal reactivation of cells was induced by infection with RTA baculovirus 48 h after transfection (negative control), and NaB and RTA baculovirus were used to induce maximal reactivation (positive control). 30 h later, the cells were analyzed for RFP expression. (B) 96 h after adding the RTA baculovirus, the supernatants from rKSHV-Vero or rKSHV-EA.hy926 were collected and used to infect U2OS target cells. 72 h after infection, the U2OS cells were analyzed for GFP expression. (C) rKSHV- EA.hy926 cells were transfected with siRNA specific for LKB1, a control siRNA (si ctrl), or left untreated (mock), and subjected to maximal reactivation (RTA+NaB) 48 h after transfection. 30 h after reactivation, the cells were analyzed for RFP expression (three bars on the left). 72 h after reactivation, supernatants were collected and used to infect näive U2OS cells. 72 h after infection, the U2OS cells were analyzed for GFP expression (three bars on the right). Values are means of two independent experiments ± SD.
