# Supplementary material for: KSHV Reactivation from Latency Requires Pim-1 and Pim-3 Kinases to Inactivate the Latency-Associated Nuclear Antigen LANA
Source: PLoS Pathog. 2009 Mar 6;5(3):e1000324. doi: 10.1371/journal.ppat.1000324 (PMC2648312; doi:10.1371/journal.ppat.1000324)
Supplement: Figure S2 — Pim-2 expression levels do not increase upon TPA treatment. BC-3, BCBL-1 as well as a KSHV-negative control cell line BJAB were treated with TPA (+) or solvent (DMSO; −) for 48 h. Whole cell extracts were immunoblotted with anti-Pim-2 antibody. Anti-tubulin served as a loading control. (0.05 MB DOC) [file ppat.1000324.s002.doc]

**Figure S2. Pim-2 expression levels do not increase upon TPA treatment.**

BC-3, BCBL-1 as well as a KSHV-negative control cell line BJAB were treated with TPA (+) or solvent (DMSO; -) for 48 h. Whole cell extracts were immunoblotted with anti-Pim-2 antibody. Anti-tubulin served as a loading control.
