# Supplementary material for: KSHV Reactivation from Latency Requires Pim-1 and Pim-3 Kinases to Inactivate the Latency-Associated Nuclear Antigen LANA
Source: PLoS Pathog. 2009 Mar 6;5(3):e1000324. doi: 10.1371/journal.ppat.1000324 (PMC2648312; doi:10.1371/journal.ppat.1000324)
Supplement: Figure S3 — Interaction of LANA and Pim-1 occurs upon induction of viral reactivation. BC-3 cells were treated with TPA (+) or solvent (DMSO; −) for 48 h. The indicated amounts of cell extracts were immunoprecipitated with anti-Pim-1 antibody and subjected to an in vitro kinase assay towards co-precipitated proteins. Samples were resolved by SDS-PAGE (8%) followed by autoradiography. The kinase filter was immunoblotted with anti-LANA and -Pim-1 antibodies. The inputs (10%) are shown on the right. (0.15 MB DOC) [file ppat.1000324.s003.doc]

**Figure S3. Interaction of LANA and Pim-1 occurs upon induction of viral reactivation.** BC-3 cells were treated with TPA (+) or solvent (DMSO; -) for 48 h. The indicated amounts of cell extracts were immunoprecipitated with anti-Pim-1 antibody and subjected to an *in vitro* kinase assay towards co-precipitated proteins. Samples were resolved by SDS-PAGE (8%) followed by autoradiography. The kinase filter was immunoblotted with anti-LANA and -Pim-1 antibodies. The inputs (10%) are shown on the right.
